# A View of Pre-mRNA Splicing from RNase R Resistant RNAs

**DOI:** 10.3390/ijms15069331

**Published:** 2014-05-26

**Authors:** Hitoshi Suzuki, Toshifumi Tsukahara

**Affiliations:** 1 Center for Nano Materials and Technology, Japan Advanced Institute of Science and Technology, 1-1 Asahidai, Nomi, Ishikawa 923-1292, Japan; 2 School of Materials Science, Japan Advanced Institute of Science and Technology, 1-1 Asahidai, Nomi, Ishikawa 923-1292, Japan; E-Mail: tukahara@jaist.ac.jp

**Keywords:** pre-mRNA splicing, RNase R, circular RNA, multiexon skipping

## Abstract

During pre-mRNA splicing, exons in the primary transcript are precisely connected to generate an mRNA. Intron lariat RNAs are formed as by-products of this process. In addition, some exonic circular RNAs (circRNAs) may also result from exon skipping as by-products. Lariat RNAs and circRNAs are both RNase R resistant RNAs. RNase R is a strong 3' to 5' exoribonuclease, which efficiently degrades linear RNAs, such as mRNAs and rRNAs; therefore, the circular parts of lariat RNAs and the circRNAs can be segregated from eukaryotic total RNAs by their RNase R resistance. Thus, RNase R resistant RNAs could provide unexplored splicing information not available from mRNAs. Analyses of these RNAs identified repeating splicing phenomena, such as re-splicing of mature mRNAs and nested splicing. Moreover, circRNA might function as microRNA sponges. There is an enormous variety of endogenous circRNAs, which are generally synthesized in cells and tissues.

## 1. RNA Digestion by RNase R Treatment

RNase R, originally identified in *Escherichia coli* [1,2], has two cold shock domains, an RNase catalytic domain, an S1 domain and a basic domain [3]. Recently, it was reported that RNase R is associated with the regulation of *trans*-translation and is regulated by transfer messenger RNA, nonstop mRNA and ribosomes [4,5,6]. Excess amounts of RNase R in a cell act as a deleterious bacterial protein. This is because RNase R is more active and more effective in degrading RNAs (even RNAs with a secondary structure) than the other bacterial exoribonucleases, such as RNase II [7]. Other than substrate RNAs that form double-stranded RNA with 3' overhangs shorter than seven nucleotides, RNase R essentially degrades all linear RNAs [8]. Key recognitions of substrate RNAs have been analyzed and the structural model of RNase R active site suggests that the 2'-hydroxyl group of the 3rd nucleobase towards the 5' from the scissile phosphate is recognized by the 463th glutamic acid of RNase R (of *Mycoplasma genitalium*) [9]. It is important for recognition of the substrate RNA, and modification of 2'-hydroxyl may affect the degradation of RNA. The biological significance of RNase R in prokaryotic cells is interesting; however, in this review, we focus on RNase R as an analytical tool for eukaryotic RNAs. Indeed, RNase R is the best 3' to 5' exoribonuclease for the methodical digestion of eukaryotic linear RNAs, although there are rare cases of RNase R resistance. In addition, mRNAs are not chemically protected at their 3' ends, unlike the protection offered by the cap structure at their 5' ends. Therefore, RNase R efficiently degrades linear mRNAs from their unprotected 3' ends.

## 2. Splicing Products as Substrates of RNase R Treatment

Eukaryotic pre-mRNA undergoes two processes during the normal splicing reaction (reviewed in [10]). The first process comprises cleavage at the 5' end of the intron and formation of a 2'–5' phosphoester bond between the branch point and its cleaved 5' end. Thus, the intermediate and 5' exon are generated. The second process comprises cleavage at the 3' end of the intron of the intermediate and the connection between the upstream and downstream exons. In addition to mRNA, an intron lariat RNA molecule is generated, with a circular region formed by a 2'–5' linkage and a linear 3' tail region.

Two methods are generally used to investigate the circular part of intron lariat RNAs. First, reverse transcriptases are able to read through the 2'–5' linkage [11], synthesizing a cDNA for the lariat product that has a junction of the branch point-5' end of the intron inside [12]. cDNA synthesis by reverse transcriptase often pauses at the 2'–5' linkage, even if it eventually reads through that region [11]. A couple of mutated, deleted or inserted nucleotides are frequently observed at the 2'–5' linkage region after reverse transcription and polymerase chain reaction (RT-PCR) and sequencing [13]. Except for the possibility of base changes, this method is simply an RT-PCR reaction that detects the intron lariat RNA.

**Figure 1:**
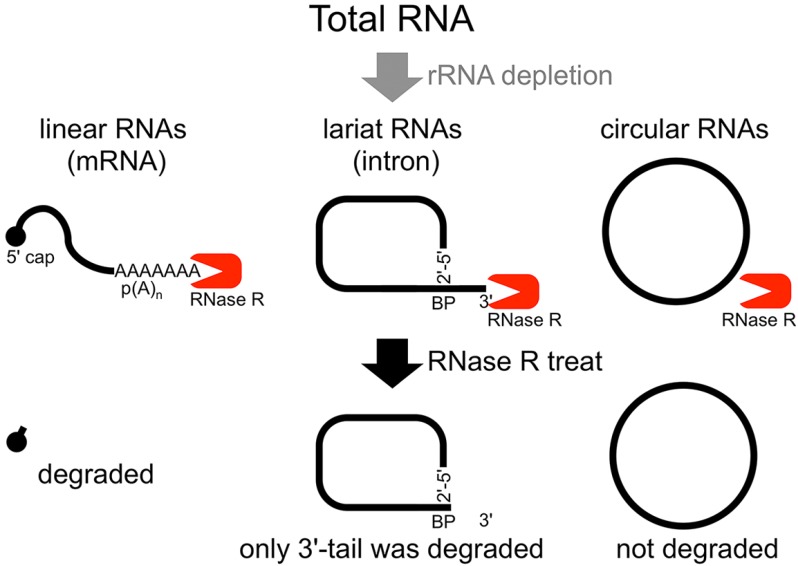
Flow chart of RNase R treatment. Linear RNAs such as mRNAs can be degraded by RNase R treatment. RNase R can also degrade the 3' tail region of an intron lariat RNA. Conversely, the circular part of the lariat RNA and circular RNAs are resistant to RNase R treatment. When total RNA is used as an RNA source, rRNA (major linear RNAs in the total RNA) depletion can help to enrich circular RNAs.

The second method of analyzing the circular structure of RNAs is RNase R treatment, which was established by our group. We showed that RNase R could not digest the circular part of an intron lariat RNA, meanwhile all other linear RNAs from the splicing reaction, including the 3' tail of the lariat RNA were digested well by RNase R [14]. In contrast, the 2'–5' linkage at the branch point of the intron lariat RNA strongly blocked RNase R digestion. Similar to *in vitro* splicing products, we showed that RNase R treatment could provide a source of circular type RNA from total RNA (Figure 1). This hypothesis was validated by RT-PCR to detect the lariat RNA [14]. In nature, a debranching enzyme hydrolyzes the 2'–5' phosphodiester at the branch point and linearizes the intron lariat RNA. Then, exoribonucleases degrade the linearized intron RNA to reuse the nucleotides. It has been suggested that rapid turnover of lariat RNAs might be important in higher eukaryotes [15]. This may explain why detection of lariat RNAs from total RNAs is quite difficult. Some circular parts of the intron lariat RNAs could be analyzed by RT-PCR and/or RNase R treatment [14], suggesting that in total RNA, lariat RNAs from the splicing reaction to the debranching reaction exist for some time. Moreover, some lariat RNAs are used as substrates to generate microRNAs (miRNA), or may be used to generate circular RNA (circRNA), as described below.

## 3. Head-to-Tail Spliced Products as RNase R Resistant RNAs

**Figure 2:**
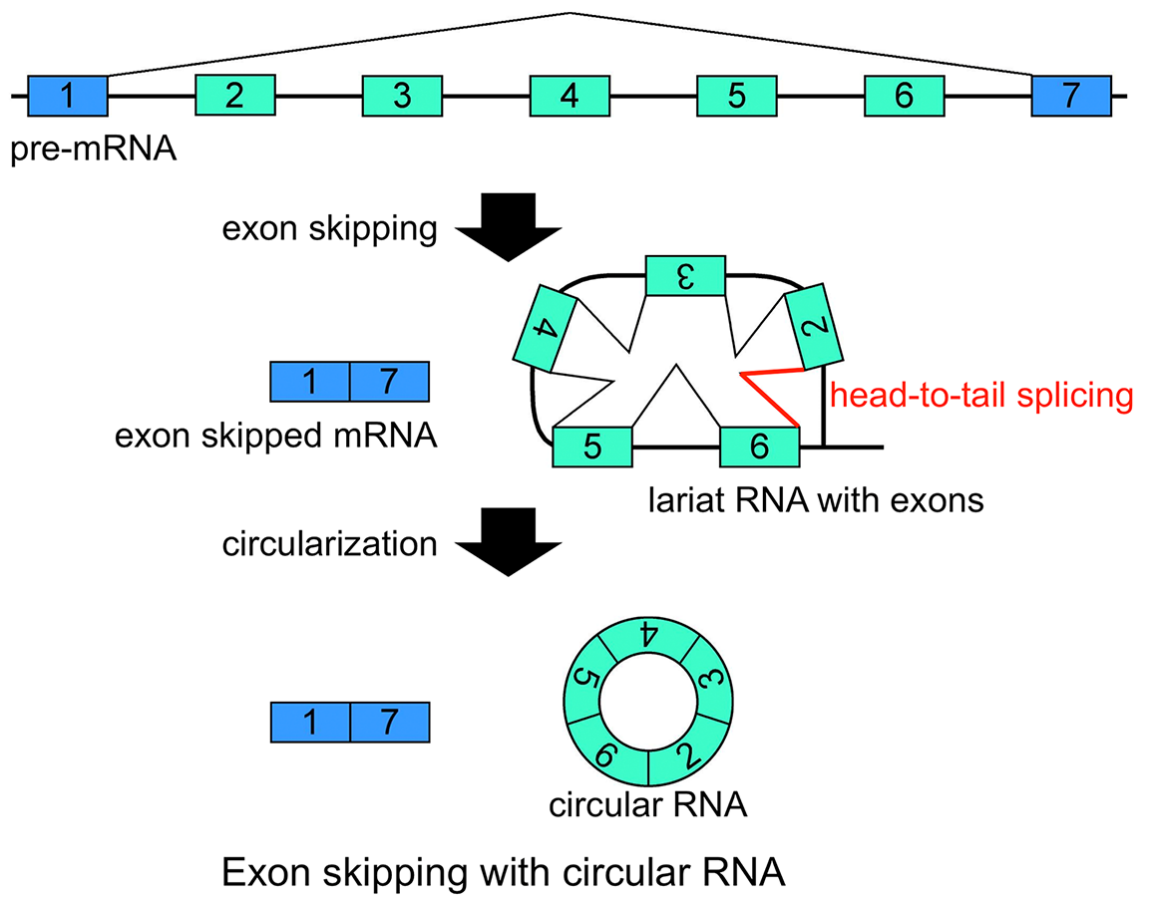
Schematic representation of a model that generates a circRNA. This is one of the most plausible models of circRNA synthesis. The red color indicates head-to-tail splicing. Although this figure is drawn to show that the conventional splicing events in intron 2 to intron 6 happen after the exon skipping event, it is possible that conventional splicing happens before exon skipping. Moreover, even when the lariat RNA contains one single exon, head-to-tail splicing could occur between a downstream donor site and an upstream acceptor site.

Another group of RNA molecules that are validated as RNase R resistant are circRNAs [14]. Structurally, circRNAs do not have 5' and 3' ends. Therefore, circRNAs are resistant to RNase R treatment (Figure 1) [14]. It was suggested that head-to-tail splicing (so-called back splicing or circle splicing), which is formed between the downstream exon/intron border (as a splicing donor site) and the upstream intron/exon border (as a splicing acceptor site), circularizes the precursor RNA [16,17,18,19,20,21]. Additionally, conventional intron(s) can be excised in temporal order, even when head-to-tail splicing is suspected. Individual analyses showed that one of the convincing precursors of the circular RNA is a lariat RNA generated by exon skipping [16,17,18,19,20,21] (Figure 2). It is thought that the 2'–5' linkage of exon(s) containing lariat RNA brings its upstream and downstream exons close enough to permit head-to-tail splicing. In addition, it was suggested recently that intronic complementary pairs, such as Alu repeat pairs, bring these exons close to generate circRNAs [22]. Alternatively, two distinct linear RNAs may become a substrate to form the circRNAs via complementary pairings [22]. Although rapid turnover is required for lariat RNAs, it is thought that some intron lariat RNAs can undergo additional splicing events between the initial splicing reaction and ultimate digestion. Thus, some circRNAs could be by-products of exon skipping events.

Historically, head-to-tail type products were reported as scrambled exons [23]. It has been reported that the scrambled products formed circular structures [24,25,26]. Moreover, some circRNAs and exon-skipped products were detected from the same RNA sources [16,17,18,19,20,21], as we described above. In addition to the models for the synthesis of circRNAs, it has been suggested that circRNAs modulate the expression of a target gene [21]. Several potential functions, such as an mRNA template of translation, a regulator of mRNA expression, and assembly and/or regulation of RNA-binding proteins, have been hypothesized (reviewed in [27]). However, their definitive physiological function was not clearly demonstrated until 2013 when Memczak *et al*. and Hansen *et al.* showed that an antisense circRNA from the cerebellar degeneration-related protein 1 transcript (CDR1as) acts as a miRNA sponge against miR-7 [28,29], thereby regulating the development of the midbrain during zebrafish embryogenesis [28]. They also showed that the circRNAs were located in the cytoplasm and formed a ribonucleoprotein (RNP) complex with miR-7 and Ago protein [28,29]. Seventy-four potential annealing sites against miR-7 are present on the CDR1as circRNA; however, the sequences of these sites only imperfectly match with the miR-7 sequence. It is possible that these imperfect base-pairings permit CDR1as circRNA to avoid being a miR target or substrate in the Dicer-RISC processes.

Recent reports of the high-throughput analyses of RNase R resistant RNAs or circRNAs showed that vast numbers of circRNAs are derived from thousands of genes in cells and tissues, much more than had been considered previously [22,28,29,30,31]. To date, it has proved very difficult to estimate accurately the amount of circRNA molecules in total RNA. Both Memczak *et al*. and Jeck *et al.* stated that certain circRNAs were enriched by >10-fold after RNase R treatment [22,28]. RNase R may not degrade linear RNAs completely. A comparison between the canonical splicing and head-to-tail splicing indicated diversity of the relative ratio among circRNAs [22]. Using their head-to-tail splicing ratio among the 7771 circRNAs (low stringency), the average of these ratios can be roughly estimated as 9.2%; although the genes generating these circRNA were listed as only 14.4% of those expressed in human fibroblast cells [22]. Furthermore, Salzman *et al.* roughly estimated that circRNA type molecules could be detected in 10% of all detectably expressed transcripts using rRNA-depleted total RNAs [30]. Based on these results, it can be roughly predicted that approximately 10% of the whole transcripts, except for rRNAs, are circRNAs. In contrast, small numbers of reads of intron lariat RNAs were observed in the high-throughput analysis of RNase R resistant RNAs [22]. Therefore, the amount of lariat RNA is not likely to affect the relative amount of circRNAs among total RNAs. However, a recent study of RNase R resistant RNAs following depletion of poly(A)+ and rRNAs identified hundreds of circular intronic long noncoding RNAs, which were similar to lariat RNAs, as major products in human cell total RNAs [31]. In addition to circRNA and lariat RNAs, Danan *et al.* showed that other types of circular RNAs, generated from permuted tRNAs, rRNA processing intermediates, or C/D box RNAs, were present in Archaea and were resistant to RNase R treatment [32].

## 4. CircRNAs and Exon Skipping

Generally, exon skipping (cassette exon) is one of five alternative splicing basic models. The other four models are mutually exclusive exons, alternative donor sites, alternative acceptor sites and intron retention. In addition, multiple promoters and multiple polyadenylation sites can produce alternative isoforms of transcripts. Sometimes, multiple alternative exons or regions are continuously located on the genome, resulting in complex alternative splicing [33]. In the case of multiexon skipping as an example of complex alternative splicing, multiple cassette exons are separated by introns and are continuously aligned between constitutive exons on the genome. These alternative exons and alternative regions can be recognized in the genome viewer by comparing them with the annotated sequences of RefSeq (reference sequence), mRNAs and ESTs. However, exons of exonic circRNAs from the registered information in the circRNA database [34] do not look like the cassette exons analyzed in the genome viewer; even some precursors of circRNAs can be generated by exon skipping. Most of these exons resemble constitutive exons. Moreover, exon skipping that generates circRNA has been referred to as alternative splicing and mis-splicing [16]. When researchers find a novel and alternatively spliced mRNA sequence, the newly joined or excised exon is generally and automatically considered as an alternative exon, even if all other data suggest that it is a constitutive exon. It is very difficult for exon skipping events involving circRNA to be simply categorized as usual and conventional “alternative splicing”, and *vice versa*. Exon skipping events in nature that result in circRNAs might not be simple mistakes or abnormal events. So far, we do not know if there are any different machineries between exon skipping with circRNAs and the usual alternative splicing. In this review, exon skipping for circRNAs is conveniently called “traceable exon skipping”.

Some exonic circRNAs are suggested to have resulted from traceable exon skipping. This is the reason why it has been assumed that a gene’s expression and its exonic circRNA expression should correlate. Among recent high-throughput analyses, different views were expressed on the relationship between circRNA expression and the expression of its gene [22,28,30]. In high-throughput analysis, the circRNAs of the low expression genes group were barely detected [22,28,30]. Meanwhile, one analysis suggested that exonic circRNAs and their canonical transcripts among human genes were expressed in a directly proportional manner [30]. In contrast, some reports stated that it was difficult to observe strong correlations between the highly expressed gene group and their exonic circRNAs [22,28]. For example, the KIAA0182 circRNA was expressed more than 10-fold higher than the canonical linear transcript from the same gene [22]. CircRNAs are more stable than canonical linear transcripts, such as mRNAs, in cells [22,28]; therefore, it is obvious that different stabilities of RNA molecules affect their expressions. Likewise, the occurrence frequency of each traceable exon skipping event and the efficiency of each head-to-tail splicing could affect the expressions of circRNAs and the canonical linear transcripts from the same gene. Thus, there may not be strong correlations between mRNA expressions and circRNA expressions.

The mechanism that regulates the stability of each circRNA molecule remains unknown. Known *cis*-acting elements that regulate the stability of linear RNAs via exoribonucleases seem to be not applicable to circularized RNA, as it is resistant to RNase R. This assumes that cleavage of circRNAs by endoribonucleases is the key for future research into circRNA stability. There is also no report on the regulatory proteins of head-to-tail splicing so far. It is thought that, for the regulatory motifs of normal splicing, such as exonic splicing enhancers, the conservation of the splice site consensus or that of the branch point [35] may influence or cause head-to-tail splicing.

It has been suggested that the head exon and the tail exon of head-to-tail splicing are located as a downstream adjacent exon and an upstream adjacent exon for the exon-exon junction of traceable multi-exon skipping in the genome view, respectively [16,17,18,19,20,21]. Therefore, it is possible that the head-to-tail spliced exons of some circRNAs, which are generally more stable than their linear transcripts, will permit the analysis of traceable multiexon skipping events as by-products. Notably, 20% of circRNA substrates perhaps have complementary ALU repeat pairs [22]. It may be possible that inner exons of the multiple-exon-containing lariat RNA can cause head-to-tail splicing instead of its most distal exon pair via an affinity for the complementary pair. Of course, the reverse transcription reaction and PCR reaction may increase any bias, whereby smaller circRNAs may be generated more efficiently than larger circRNA in the same reaction. In principle, circRNA comprising a large number of exons has one single head-to-tail spliced junction and more canonical splicing junctions. Therefore, it is likely that the head-to-tail sequence of smaller circRNAs would be detected more frequently than that of larger circRNAs. Although such influences should be considered, high-throughput analysis showed that the number of exons in circRNAs was typically one to five [28]. In addition, it is thought that frequently detected junction exon(s) of the head-to-tail splicing could represent the excised exon(s) in a traceable multiexon skipping event. Meanwhile, it was reported that a defect in the nonsense mediated mRNA decay (NMD) pathway increased aberrant splicing events in 30% of all expressed genes [36]. The data also showed that ablation of NMD in the mouse liver generated isoforms containing premature termination codons and other isoforms, among which were approximately 3000 exon skipping events, consisting of single and multiple exon skipping events, and approximately 2000 other splicing events [36]. Indeed, abundant splicing error events, such as traceable multiexon skipping, naturally occur in cells and tissues and are degraded by NMD.

**Figure 3:**
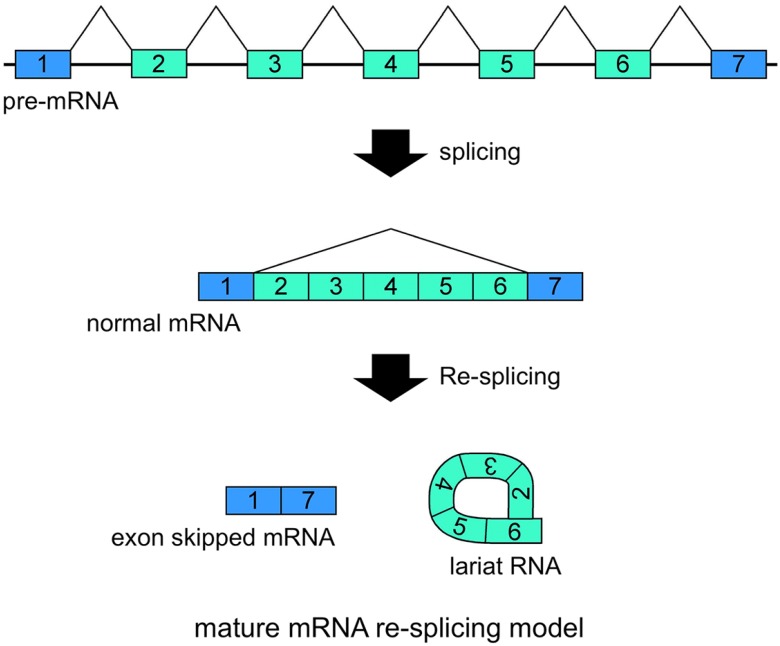
Schematic representation of the mature mRNA re-splicing model. Correlated expressions of exon skipped mRNA and exonic lariat RNA (lariat exon) indicated that this splicing model operates for the *TSG101* (tumor susceptibility gene 101) and *FHIT* (fragile histidine triad protein) genes [37]. The first splicing conventionally generates normal mRNA. Although the spliced products are not usually involved in additional splicing processes, normal *TSG101* and *FHIT* mRNAs are probably involved in the second splicing events. Re-splicing of mature mRNA produces multiple exon skipped mRNAs and exonic lariat RNAs.

## 5. Exonic Lariat RNAs and Exon Skipping

CircRNAs are naturally stable and more abundant in cells and tissues than was thought previously. In addition, the ablation of NMD suggested that traceable exon skipping-type events happen frequently in cells. Although several processes, from the generation of exon skipping to the degradation of circRNAs, may make the structures and proportions of circRNAs inaccurate to indicate the occurrences of the traceable multiexon skipping events, certain exonic circRNAs will help to analyze traceable exon skipping as by-products.

**Figure 4:**
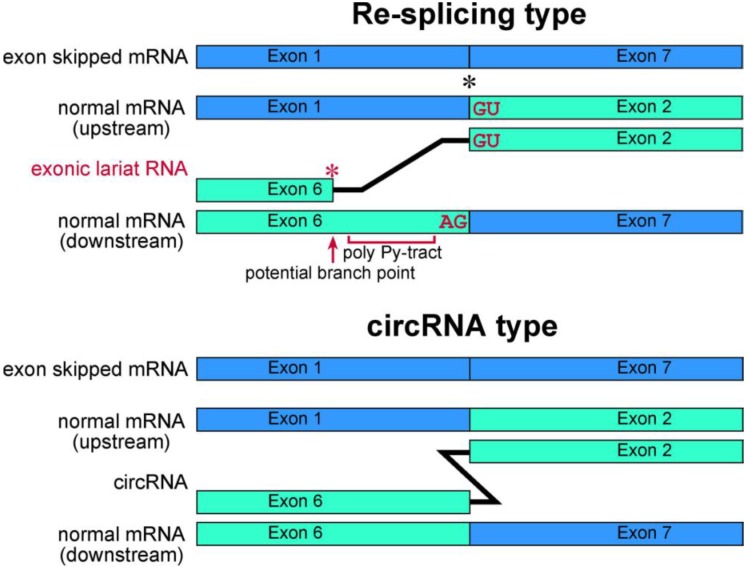
Schematic representation of exonic lariat RNA and circRNA. The mature mRNA re-splicing produces exonic lariat RNA. Therefore RT-PCR can amplify a product that lacks a potential poly pyrimidine-tract region between the potential branch point and 5' splice site of the re-splicing. The splice site consensus; GU and AG, should be required for the re-splicing. A red asterisk indicates frequent occurrence of the base substitutions [13]. Meanwhile, these features are not applicable in circRNA. Instead, the head-to-tail splicing should precisely connect exons of circRNA. As shown by black asterisk, an exon-exon junction of normal mRNA is used as the splice site in *FHIT* re-splicing event. However, it is possible that the splice sites of the re-splicing are located in the middle of exons in normal mRNA, such as *TSG101* [37].

Our recent research has involved investigating lariat RNAs, which are RNase R resistant by-products of splicing events. Our group reported another splicing model for skipping multiple exons that essentially annotated constitutive exons in the *TSG101* and *FHIT* genes [37]. Their transcripts are abnormally spliced in cancer cells and form multiexon skipped mRNAs (Figure 3). Assuming possible and potential splicing, splicing by-products were analyzed by RNase R treatment and RT-PCR experiments. Exonic lariat RNA (lariat exon) type products were found as RNase R resistant RNAs, but not circRNAs. The detected products had the structural features of general lariat RNAs, such as a connection between GU sequences at the 5' end and a hypothetical branch point [37]. These branch points are located approximately 20 nt upstream of the acceptor site of the multiexon skipping on the normal mRNAs. Although cDNA products from lariat RNAs and circRNAs can be synthesized and amplified by RT-PCR, the precise feature of these two kinds of RNA molecule groups can be detected in the sequences of skipped products (Figure 4). Thus, exonic lariat products of *TSG101* and *FHIT* genes are clearly different from circRNA products, which are connected by head-to-tail splicing. Indeed, these results suggested that normal mRNAs generated by the first splicing step were used as the substrate for the second splicing (Figure 3). Multiexon skipped mRNAs and exonic lariat RNAs could be produced in cancer cells, where it may be termed mature mRNA re-splicing [37]. There are some problems with this re-splicing hypothesis. For example, the exon-exon junction complexes (EJCs), perhaps formed in the first splicing and that repress subsequent splicing events, may need to be removed for the second splicing. However, re-splicing of mature mRNAs has some advantages. For example, the initial constitutive splicing events greatly shorten the distance between activated splice sites to generate smaller mRNAs.

Shortening of the intron may activate conventional splicing in an extremely long intron. We investigated a splicing event in *DMD* (Duchenne muscular dystrophy) intron 7, whose length is 110,119 nt, using RT-PCR and RNase R treatment in total RNA from human skeletal muscle tissues and cells [38]. We searched for splicing by-products produced from the many combinations of potential splice sites inside the intron. Ultimately, two small lariat RNA type products were detected deep inside the intron. These products were co-transcriptionally expressed in terminal muscular cell differentiation [38]. In short, these results suggested that the nested-splicing model produces small splicing events inside of an intron, which shorten the long intron and activate the final conventional splicing. The proximal, smaller splicing events perhaps shorten the length of the introns and activate the next splicing step; thus, these multi-step splicing models, such as re-splicing of mature mRNAs and nested splicing, may have common mechanisms.

As mentioned above, we proposed two splicing models (re-splicing and nested splicing), based on analysis of lariat RNAs. RNase R resistant RNAs may provide valuable information about splicing processes that are not revealed by analysis of mRNAs. Major cellular RNase R resistant RNAs, the circRNAs, are also informative for splicing events, even though some circRNAs may not match with the skipping events. In addition to their merits as splicing markers, circRNAs have their own molecular functions. For instance, CDR1as circRNAs act as miRNA sponges for miR-7 and are related to brain development [28]. Similar to long non-coding RNAs, circRNAs may be associated with various biological functions. Thus, RNase R resistant RNAs may represent an as-yet unexplored level of functional RNAs.

## 6. Conclusions

RNase R is an ideal enzyme to isolate the circular parts of lariat RNAs and circRNAs. In addition to these two types of RNAs, circRNAs from premature tRNAs, rRNA intermediates and C/D box RNAs are also RNase R resistant. RNase R treatment represents a new tool to analyze non-linear RNAs. Validation of RNase R resistant RNAs will help to clarify natural splicing processes and repetitive splicing events. In addition, RNase R contributes to the analysis of the molecular functions of circRNAs. Thus, RNase R treatment is the key to opening up the next RNA frontier.
